# Leveraging spatial data infrastructure for machine learning based building energy performance prediction

**DOI:** 10.1371/journal.pone.0335531

**Published:** 2025-10-27

**Authors:** Suleyman Sisman, Abdullah Kara, Arif Cagdas Aydinoglu

**Affiliations:** 1 Department of Geomatics Engineering, Gebze Technical University, Kocaeli, Türkiye; 2 Faculty of Architecture and the Built Environment, Delft University of Technology, Delft, The Netherlands; The Hong Kong Polytechnic University, HONG KONG

## Abstract

The calculation, management and maintenance of energy performance of buildings (EPBs) are significant in increasing energy efficiency in buildings and reducing greenhouse gas emissions since it is estimated that approximately one third of energy consumption is associated with buildings, and furthermore, three-quarters of the existing building stock is characterized by energy inefficiency. However, in many cases, EPBs are either not calculated or not integrated in a register within national spatial data infrastructure (NSDI). This complicates policy development and planning for both local and national governments, which may result in numerous complications. The objective of this paper is twofold: firstly, to design a building energy data model as an extension of NSDI in Türkiye and then implementing and populating it with real data taken from energy performance certificates from the Tuzla District in Istanbul; and secondly, to develop energy performance prediction models with Machine Learning (ML) algorithms (i.e., Random Forest (RF), Gradient Boosting Machine (GBM), Light Gradient Boosting Machine (LightGBM) and Extreme Gradient Boosting (XGBoost) in order to estimate the overall performance scores of the buildings. The model’s findings demonstrated robust predictive accuracy, achieving an R² of 0.818 (XGBoost) and performance metrics of RMSE = 5.153, MAE = 2.886, and MAPE = 3.369. These results substantiate the model’s reliability in estimating targeted building energy performance scores. These predictions can be used to provide a comprehensive overview of districts in terms of EPB and inform the development of road maps at the district, city, or national level. Furthermore, the predictions can support the development of EPB-related legislation, facilitate the design of incentive and sanction mechanisms, and promote broader sustainability and climate mitigation goals in a practical manner. Nevertheless, as a limitation of this study, the model has only been tested in a single district, which restricts its generalizability; it should therefore be evaluated in other areas to confirm its applicability.

## Introduction

In Western societies, most people spend more than 80–90% of their day inside buildings [1]. The primary purpose of buildings is to provide the basic structures necessary for human life. They provide space for a variety of activities, including housing, commercial operations, industrial production, education, healthcare, cultural gatherings, and more. The basic daily activities within these buildings, such as heating and cooling, water heating, lighting, cooking, and operating appliances and equipment, require significant energy consumption to meet the occupants’ needs. According to the International Energy Agency (IEA), buildings account for 30% of global final energy consumption [2], while the European Commission states that they account for 40% of total energy consumption in the European Union (EU) [3]. In addition, while it is estimated that 80% of the energy used in EU homes, and that over one-third of the EU’s energy-related greenhouse gas (GHG) emissions originate from buildings, roughly 75% of the EU building stock is considered energy inefficient [3].

It is imperative to periodically calculate/measure, identify, manage and maintain the energy performance of buildings (EPB) to formulate a roadmap for enhancing their efficiency and reducing GHGs emissions. Indeed, EPB has been accentuated by numerous international guiding references and frameworks in recent years.

### International and regional guiding references and frameworks on energy performance of building and energy performance certificate

Despite the absence of explicit reference to the EPB in the United Nations (UN) Sustainable Development Goals (SDGs), the goals address related concerns through a multitude of interconnected objectives, including affordable and clean energy (SDG 7), sustainable cities and communities (SDG 11), responsible consumption and production (SDG 12), and climate action (SDG 13). It is evident that several particular objectives serve to underscore the significance of enhancing the energy performance of buildings, albeit in a somewhat indirect manner. As articulated in SDG 7.3, there is an imperative to “*double the global rate of improvement in energy efficiency*”. Similarly, SDG 11.6 underscores the necessity of “*reduce the adverse per capita environmental impact of cities*”. In addition, target 12.c of the SDGs calls for the rationalization of “*inefficient fossil-fuel subsidies that encourage wasteful consumption*” while indicator 13.2.1 promotes the development of strategies that “*foster climate resilience and low greenhouse gas emissions development*” [4]. Furthermore, the New Urban Agenda (NUA) of the United Nations Human Settlements Programme (UN HABITAT) supports and promotes the implementation of building codes and standards, as well as the retrofitting of existing buildings to improve performance. This is particularly evident in paragraph 75, which encourages governments “*to develop sustainable, renewable and affordable energy and energy-efficient buildings and construction modes and to promoting energy conservation and efficiency, which are essential to enable the reduction of greenhouse gas and black carbon emissions*” [5]. The Energy Efficiency Standards in Buildings of the United Nations Economic Commission for Europe (UNECE) have emphasized the significance of the management of EPB, and it is stated that governments must implement mandatory building energy codes and establish a pathway to ensure their new building codes and standards are performance-based and aimed at achieving zero carbon across the entire lifecycle of a building as expeditiously as possible [6,7]. Furthermore, it is indicated that the maintenance of certification of buildings to ensure energy performance must be maintained throughout their lifecycle [6]. The International Finance Corporation (IFC), a sister organization of the World Bank and member of the World Bank Group, *Green Buildings* has indicated that “*Labeling and energy performance certifications for buildings and appliances help ensure compliance with green standards, and help investors measure, verify, and compare their green building investments*” [8]. The Global Alliance for Buildings and Construction (GlobalABC)‘s Roadmap for Buildings and Construction, prepared by the IEA, emphasized that the implementation of building energy certification and labelling can facilitate the disclosure of existing building performance through benchmarking and evaluation, thereby enabling the enforcement of performance requirements [9]. Additionally, the utilization of building passports can enable the tracking of information regarding the building, materials, systems, energy use, renovations, and other pertinent data, thereby enhancing the decision-making processes through the improvement of data quality, tracking, and storage [9].

In addition to the international guidance documents, the International Organization for Standardization (ISO) published a series of standards for the assessment of EPB in 2017, designated ISO 52000. This set of standards encompasses calculation methods for EPB, requisite input data for calculations, the content of building energy certificates (e.g., ISO 52000−1, ISO 52003−1 and ISO 52003−2), graphical representations of energy ratings and quality control of numerical indicators, rating requirements and certificates [10–12]. Various energy performance rating systems exist around the world, such as Energy Star in the United States, the National Australian Built Environment Rating System (NABERS), and the energy performance certificate in the EU. The ISO 52000 series of standards can be used to benchmark these rating systems and is utilised by numerous parties as a foundation for structuring, building or updating regional and national EPB standards and rating systems including energy performance certificates. For instance, the European Union (EU)‘s Energy Performance of Buildings Directive (EPBD), which was formally introduced in December 2002 and most recently revised in May 2024, indicates that “*Member States shall describe their national calculation methodology on the basis of Annex A to the key European standards on the energy performance of buildings, namely (EN) ISO 52000-1, (EN) ISO 52003-1, (EN) ISO 52010-1, (EN) ISO 52016-1, (EN) ISO 52018-1, (EN) ISO 52120-1, EN 16798-1 and EN 17423 or superseding documents*” [13]. EPB is defined by this directive as “*calculated or metered amount of energy needed to meet the energy demand associated with a typical use of the building, which includes energy used for heating, cooling, ventilation, domestic hot water and lighting*” [13]. The distinction between new and existing buildings with regard to the issuance of an energy performance certificate is a matter of significance in the directive. In the case of newly constructed buildings or building units, the certificate is mandatory. Similarly, in instances where an existing building undergoes significant renovation, or is sold, rented out, owned or occupied by public bodies, the certificate is also mandatory [13]. As outlined in the directive, the content of an energy performance certificate is typically comprised of the following elements: (a) the energy performance class, ranging from A to G, (b) the calculated annual primary and final energy use and consumption, (c) the operational greenhouse gas emissions, (d) recommendations for improvements in energy efficiency, (e) the validity of the calculations and a maximum deviation for the energy performance, (f) validity check of the input data of the building (e.g., floor area, year of construction, renovation status, construction type, energy source type) and so on. Furthermore, the EPBD has also clearly emphasized the importance of setting up “*a national database for the energy performance of buildings*” and populating it with real data “*to be gathered from all relevant sources related to energy performance certificates*” [13].

EPBD is utilised by numerous directives including the INfrastructure for SPatial Information (INSPIRE) directive which aims to establish an EU Spatial Data Infrastructure (SDI) [14]. The objective of INSPIRE is to facilitate the exchange of environmental spatial information among public sector organizations and to enable public access to such information [14]. The INSPIRE directive addresses 34 spatial data themes required for environmental applications, one of which is the Building Data Theme [15]. This theme includes a data type designated for recording energy performance (i.e., EnergyPerformance), which encompasses the attributes energyPerformanceValue (the class of energy performance, ranging from A to G), dateOfAssessment (the date when the energy performance was assessed) and assessmentMethod (the reference to the document describing the assessment method of energy performance) [15]. The implementation of INSPIRE data themes has been adopted across a diverse range of EU member states, as well as a number of non-EU member states, including Türkiye, with the objective of facilitating the development of a national SDI (NSDI). The following subsection will present the situation in Türkiye by first introducing the current state of energy performance in buildings and energy performance certifications, as well as their inclusion in the National Spatial Data Infrastructure (NSDI).

### Background on energy performance certificate in Türkiye and predictive modelling of EPB in literature

Mandatory certification and rating (scoring or labelling) of buildings has been introduced in various countries with the aim of achieving a certain level of energy efficiency. One of these countries is Türkiye. In 2007, Energy Efficiency Law No. 5627 was enacted, introducing the energy performance certificate and its minimum content, which includes information on the building’s energy needs, insulation, efficiency of its heating and/or cooling systems, and its energy consumption class [16]. One year later, the implementing regulation of this law was enacted with the name “Regulation on Energy Performance in Buildings” [17]. This regulation aims to ensure the effective and efficient use of energy and energy resources in buildings, prevent energy waste and protect the environment, and is based on the EPBD [18]. This regulation, which has been amended several times since its issue date, specifies the content and calculation of the energy performance certificate, the mandatory conditions and the starting date for new and existing buildings, as well as the validity period and the organizations responsible for issuing the energy performance certificate. In addition to the content determined in the law, the regulation states that the following information should also be included in the energy performance certificate: (a) general information about the building (national building identifier, number of floor, height of floors), (b) information about the organization and the responsible person who issued the energy performance certificate, (c) gross and net area of the building, (d) purpose of use of the building (e.g., residential apartment, government property), (e) amount of energy used for heating, cooling, air conditioning, ventilation and hot water supply of the building (kWh/year), (f) annual primary energy amount according to each type of energy consumed (kWh/year), (g) classification of annual primary energy consumption per usage area of the building according to a reference scale ranging from A to G, (h) annual amount of GHG generated by final energy consumption per usage area (kg CO₂/m²-year), (i) classification of annual GHG emissions per usage area of the building according to a reference scale ranging from A to G (kg CO₂/m²-year), (j) lighting energy consumption value of the building, (k) energy class according to primary energy consumption, (l) CO₂ emission class according to final energy consumption and (m) renewable energy usage rate [17].

The regulation’s related article was implemented on 1 January 2011, marking the start of the requirement for newly constructed buildings to obtain an energy performance certificate with at least Class C energy consumption and carbon dioxide emissions. Buildings without an energy performance certificate have not been granted an occupancy permit since then [18]. Existing buildings with an occupancy permit issued before 1 January 2011 are also obliged to obtain an energy performance certificate. From 1 January 2020 onwards, an energy performance certificate has been mandatory for all buildings subject to sale or rental transactions, although there are some exceptions, such as industrial, agricultural, religious, protected, temporary buildings and buildings outside urban areas with a total construction area of less than 1,000 m², and so on [17]. It is important to note that the energy performance certificate must be prepared for the entire building, not for building parts [18].

An energy performance certificate must include calculated information on the building’s energy requirements and energy consumption classification, insulation, and the efficiency of heating and/or cooling systems, at minimum. These calculations are performed using the Building Energy Performance Calculation Method Software (BEP-TR) for existing and new buildings, and this software can be accessed via the website of the Ministry of Environment, Urbanization and Climate Change. Companies that provide energy-efficiency consultancy services and are approved under Law No. 5627 are allowed to issue energy performance certificates and these companies must have staff who have successfully completed energy performance certificate regulation training. In addition, the energy performance certificate is valid for 10 years from the date of issue [18].

The total number of energy performance certificates is important information for creating road maps for improving the energy efficiency of buildings. As indicated by data provided by the Ministry of Environment, Urbanization and Climate Change in July 2023, energy performance certificates have been obtained for 1,500,000 buildings, of which 1,160,000 are newly constructed and 340,000 represent existing buildings [18,19]. According to the 2020 statistics of the Turkish Statistical Institute (TurkStat), the total number of buildings in use in Türkiye is 11,598,446 [20]. The exact number of energy performance certificate-ready buildings remains unknown; however, it is estimated that approximately 9 million of these buildings are constructed for residential purposes [20]. Given the fact that an energy performance certificate is mandatory for residential buildings in urban areas, it can be stated that a significant proportion of buildings are not in possession of an energy performance certificate.

On the other hand, the importance of EPB in recognized in various strategic plans in Türkiye. For instance, “The Strategic Plan (2024-2028)” has been prepared by the Strategy and Budget Presidency of Türkiye, and it has been indicated therein that investments which will increase energy efficiency will be supported, to create living spaces that are resistant to disasters, and sustainable environments. Furthermore, the “Energy Efficiency 2030 Strategy and II. National Energy Efficiency Action Plan (2024-2030)”, which was prepared by the Ministry of Energy and Natural Resources, includes the following measures: (a) the establishment of systems in accordance with ISO 50001 standards, (b) the conducting of monitoring and auditing for energy performance certificate, (c) the inclusion of the energy performance certificate information of the building in real estate advertisements, (d) the ensuring of awareness-raising by cooperating with non-governmental organizations in the real estate sector, and (e) the taking of steps to support residences with energy performance certificate in existing buildings with financial incentives, for example by offering higher mortgages.

The development of the NSDI in Türkiye has been influenced by the EPB-related legislation. Specifically, the Turkish National Geographic Information System (TUCBS) is an NSDI that has been established to facilitate the sharing and management of geographic data in a standardised manner. In TUCBS, the development of geographical data standards for various themes, including buildings, and the development of data sharing legislation and methods are included [21]. The information included in the energy performance certificate is partly incorporated within the Building Data Theme of TUCBS [22]. Notably, energy performance certificate is not documented as a source document in the building data model, which lacks certain information elements, such as energy consumption data (e.g., hot water, cooling and heating), the overall energy performance score and the amount of GHG generated by final energy consumption. Furthermore, the energy performance certificate-related data, including issue data, expiration date, issued party, and issued party identifier, as designated by the relevant ministry, are not encompassed within the scope of the building data model of TUCBS.

As previously mentioned, the majority of buildings in Türkiye do not currently have an energy performance certificate. This absence hinders the planning of an energy-efficient built environment for sustainable cities. A plethora of studies have been conducted in the literature to estimate EPB and energy consumption prediction. For instance, [23] proposes the use of Geographic Information System (GIS)-based building energy modelling to support the development of urban energy plans, with the aim of minimizing overall energy consumption and GHG emissions across the building stock. The researchers utilised existing energy performance certificate-building data to predict the energy performance of buildings lacking an energy performance certificate and the prediction was made with GIS-based building energy modelling employing Machine-Learning (ML) models. [24] investigates the suitability of ML algorithms (e.g., Random Forest (RF), Decision Tree (DT), Gradient Boosting Machine (GBM) and so on) for predicting annual building energy consumption using a large dataset of residential buildings at the early design phase to reduce the construction of more energy-inefficient buildings. [25] employs the residential energy consumption survey dataset and three tree-based ML algorithms (i.e., Light Gradient Boosting Machine (LightGBM), Extreme Gradient Boosting (XGBoost)) to develop separate energy use intensity prediction models for residential buildings. The findings indicate that the LightGBM-based prediction model demonstrates optimal performance for the prediction of apartments. Furthermore, SHAPley Additive exPlanations (SHAP) was applied in order to analyse the impact of household features on energy consumption, with the results indicating that total square footage, space heating with natural gas, climate conditions and building age are key features influencing energy use intensity. [26] asserts that ML models are a more popular option for the prediction of energy consumption, with regression tree, RF, Support Vector Regression (SVR), Artificial Neural Networks (ANNs), and ensemble models being notable examples.

Building Information Modelling (BIM)-based solution is also proposed for improving EPB. For instance, [27] emphasizes that the effective implementation of sustainable building performance is contingent upon the integration of knowledge management systems, namely an integrated knowledge-based building management system that employs multiple dimensional BIM applications, to efficaciously manage and disseminate building maintenance information throughout the post-construction lifecycle. [28] explores the potential of BIM to enhance the accuracy, reliability, and efficiency of energy labelling. The study also examines how BIM can assist in mitigating the performance gap through data updates, enhanced interoperability, and more sophisticated occupant behaviour modelling. The findings have the potential to improve current labelling methods, support broader sustainability goals, and enable stakeholders to use BIM for more accurate energy labels. Last but not least, [29] puts forward the suggestion of incorporating building passports, encompassing owner information and cadastral link, three-dimensional location, materials, certifications, life cycle assessment, pricing and economic value, into land administration systems as an element of NSDI.

### Problem, aim and methodology

**Fig 1:**
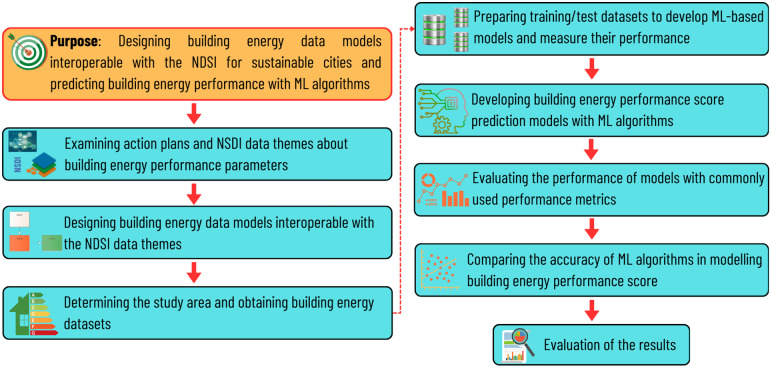
The research steps followed.

In recent years, significant progress has been made in the realm of EPB, with numerous initiatives being undertaken to facilitate the calculation, documentation, management and maintenance of EPB on a global scale. A significant number of countries have enacted legislation mandating the use of energy performance certificates with the objective of enhancing energy efficiency and reducing GHG emissions. A notable example of this trend is Türkiye, where the implementation of energy performance certificates has been mandatory for over almost two decades. However, given that many buildings were constructed prior to the introduction of energy performance certificate legislation, a significant proportion of buildings in Türkiye do not have an energy performance certificate. This situation gives rise to deficiencies in the development and planning of sustainable and climate-adaptive policy at both the local and national levels. In order to overcome this issue, the establishment of a national database populated with data gathered from energy performance certificates can be considered a preliminary step. Such a database would be capable of recording, managing and maintaining energy performance certificate-related data, as well as determining buildings lacking energy performance certificate. Furthermore, such a database should be integrated into NSDI and associated with other datasets, including address, cadastral parcel and building data (and 3D building data), in order to support efficient decision-making processes. On the other hand, such a database can be used as a source to predict EPB with various ML algorithms, as has been demonstrated in many studies in literature. Although many studies in literature have focused on predicting EPB and improving the management of EPB information, no study has been found that harmonizes EPB prediction through NSDI information in a holistic way. Therefore, this study endeavours to conceptualize a building energy data model as an augmentation of NSDI in Türkiye, followed by its implementation and populating with authentic data derived from energy performance certificates from the Tuzla District in Istanbul. Secondly, the recorded datasets relate to GIS and are used to develop energy performance prediction models with ensemble learning algorithms (i.e., RF, GBM, LightGBM and XGBoost) in order to estimate the overall performance scores of buildings lacking energy performance certificates. In other words, this study seeks to answer the question: Can machine learning models predict EPB using spatially integrated data? The research steps to achieve these goals are given in Fig 1.

The remainder of the paper is organized as follows: Section 2 presents how the NSDI of Türkiye is extended with information gathered from energy performance certificates. Section 3 provides information on the case study area and introduces the GIS-based database containing building and energy performance certificate information. It also provides details of the ensemble learning algorithms to be used to predict the overall energy performance score of buildings lacking an energy performance certificate. Section 4 presents the analysis results and evaluation. The final section provides a brief discussion and concludes the paper.

### Extending NSDI with information in energy performance certificate

**Fig 2:**
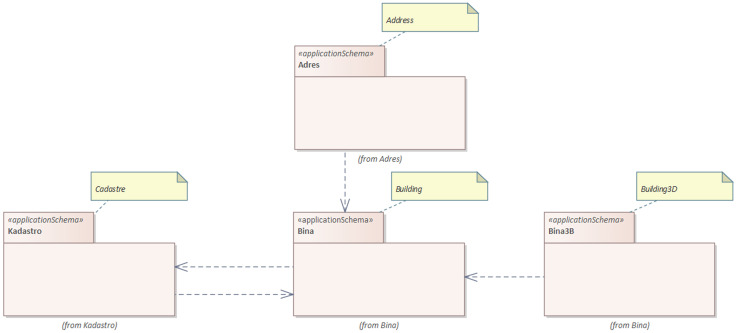
The package level relationships in NSDI of Türkiye (TUCBS).

The development of an NSDI has a history of more than two decades in Türkiye. This process was formalized with the 2019 Presidential Decree on Geographical Information Systems [30]. The purpose of this decree is to produce, manage, use, access, secure, share and distribute geographic data within TUCBS [30]. The promotion of data interoperability among providers and users is facilitated by TUCBS, which consists of 32 geographic data themes and 52 sub-data themes [31]. It aligns with INSPIRE specifications and meets national user requirements [31]. TUCBS also specifies the interrelationships between different data themes, such as cadastre, addresses and buildings (including 3D buildings), as shown in Fig 2. These relationships enable the integration of different data models, which could facilitate standards-based interoperability and cross-data model decision-making.

**Fig 3:**
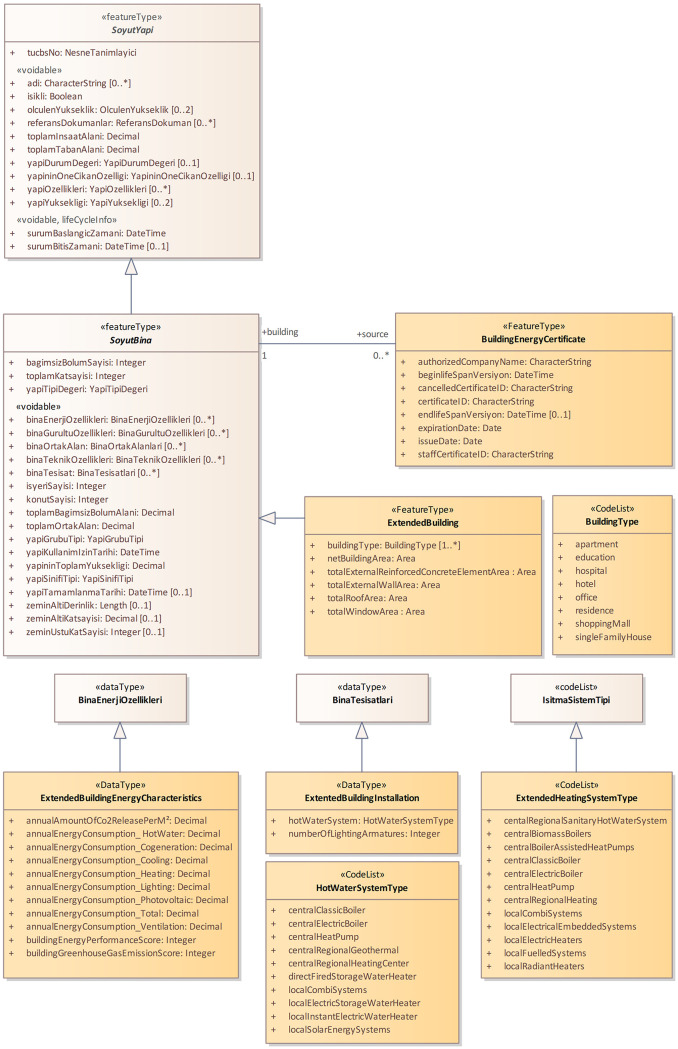
The building data model extended with the information obtained energy performance certificates. Orange classes presenting the extension.

One of the data themes in TUCBS is building. Energy efficiency and the management of energy resources were taken into consideration during the development of the data model for the building data theme [22]. However, not all the information included in energy performance certificates are included in the building data model. To elaborate further, the building data model has a class Abstract Structure (SoyutYapı), which includes total building area (toplamInsaatAlani) attribute is also included in energy performance certificates. In addition, the number of floors (toplamKatSayisi), number of floors underground (zeminAltiKatSayisi) and number of floors above ground (zeminUstuKatSayisi) attributes, which are included in the energy performance certificate, are also part of the Abstract Building (SoyutBina) class, see Fig 3. The BuildingEnergyCharacteristics (binaEnerjiOzellikleri) data type, which is defined within the Abstract Building class, encompasses the following information: (a) energy consumption class from A to G (i.e., lighting, ventilation, cooling, heating), (b) GHG emissions class from A to G, (c) total conditioned floor area of building, (d) renewable energy ratio of building and so on. Furthermore, ‘Abstract Building’ also encompasses data types, building technical characteristics (binaTeknikOzellikleri) and building installation (binaTesisat), see Fig 3. The former includes information on roof type (catiTipi), while the latter compasses the lighting system (aydinlatmaSistemi), ventilation system (havalandirmaSistemi), cooling system (sogutmaSistemi) and heating system (isitmaSistemi) attributes, which are incorporated directly into energy performance certificates.

Whilst TUCBS building data model incorporates data on effective energy management in buildings, it does not include certain key elements recorded in the energy performance certificate. The present study proposes an extension to the building data model, with the objective of developing a comprehensive data infrastructure for the management of building energy performance. The extension model was developed as an integrated component of the building data model and the existing relationships within TUCBS being preserved. The extension includes two new classes: BuildingEnergyCertificate and ExtendedBuilding.

BuildingEnergyCertificate class, which is associated with the Abstract Building, includes energy performance certificate document related information that is not included in TUCBS building data model (Fig 3). In this particular context, the following attributes are included in BuildingEnergyCertificate: (a) the date of energy performance certificate is issued (issueDate), (b) the expiration date of energy performance certificate (expirationDate), (c) the identifier of energy performance certificate (certificateID), (d) the certificate number of the individual who issued energy performance certificate (staffCertificateID), (e) the name of the company with which the individual is affiliated (authorizedCompanyName), (f) the database time management (beginLifespanVersion and EndLifespanVersion) and (g) the cancellation of energy performance certificate due to changes in structure or misleading information (canceledCertificateID). It is important to note that a building may have multiple energy performance certificates, and the legislation stipulates that the validity period of an energy performance certificate is ten years. It is therefore evident that zero or more multiplicity is defined on the BuildingEnergyCertificate side of the association between BuildingEnergyCertificate and AbstractBuilding. The incorporation of such information into TUCBS has the potential to facilitate the process of querying and managing energy performance certificate-related information. Furthermore, it can assist decision-makers in obtaining a comprehensive understanding of the situation of EPB. Furthermore, owners of buildings can be notified of the imminent expiration of the energy performance certificate, which may motivate them to undertake the process of obtaining a new certificate.

The ExtendedAbstractBuilding class, which is created as a subclass of the Abstract Building (SoyutBina) class, includes (a) the buildingType attribute, which can be different building types defined in energy performance certificates such as apartment, detached house, education, and hospital (see BuildingType code list in Fig 3), (b) the total net floor area of building (netBuildingArea), (c) the total roof area (totalRoofArea), (d) the total external reinforced concrete area used in building (totalExternalReinforcedConcreteElementArea), (e) the total external wall area of building (totalExternalWallArea), (f) the total window area of building (totalWindowArea).

In the extension model, the creation of the ExtendedBuildingEnergyCharacteristics and ExtendedBuildingInstallation data types, which are related to the Abstract Building, is essential for the comprehensive inclusion of all energy performance certificate information. ExtendedBuildingEnergyCharacteristics data type includes (a) annual total (overall) energy consumption (buildingEnergyPerformanceScore), (b) the building’s GHG emission score (buildingGreenhouseGasEmissionScore), (c) the building’s annual CO_2_ emission amount per m^2^ (annualCO2EmissionAmountPerM2), as well as the building’s energy consumption according to (d) heating (annualEnergyConsumption_Heating), (e) sanitary hot water (annualEnergyConsumption_HotWater), (f) cooling (annualEnergyConsumption_Cooling), (g) ventilation (annualEnergyConsumption_Ventilation), (h) lighting (annualEnergyConsumption_Lighting), (i) cogeneration (annualEnergyConsumption_Cogeneration), (j) photovoltaic (annualEnergyConsumption_Photovoltaic) and annual total energy consumption (annualEnergyConsumption_Total).

ExtendedBuildingInstallation, on the other hand, includes the attributes for (a) the total number of armatures in the building’s lighting system (numberOfLightingArmatures) and (b) the hot water system (hotWaterSystem), which refers to the systems that facilitate the elevation of water at network temperature to the requisite temperature for utilization within the building. Lastly, two additional code lists, HotWaterSystemType and ExtendedHeatingSystemType, are incorporated within the energy extension model. The former has been developed for the purpose of incorporating all individual and central water system types within the model, whereas the latter has been designed for the inclusion of all individual and central heating system types.

The proposed extension can enable the integration of more qualified building energy data, while ensuring compatibility with the existing TUCBS data model. The extended model has the capacity to facilitate the management of meaningful data through spatial and ML analyses, thereby enhancing the efficacy of building energy management. Additionally, it may play a pivotal role in the development of energy efficiency applications.

## Materials and methods

The developed EPB extension model to TUCBS can be used to develop a geodatabase, which can be used to predict the overall energy performance score of buildings using ML algorithms. In order to perform this task, next subsection introduces the case study area, the datasets and the created GIS system and geodatabase, developed using the EPB extension model to TUCBS. Later, the reader will find a brief overview of the decision tree-based ensemble learning algorithms that have been utilised. Finally, in this section, the performance metrics that have been employed in this paper are outlined.

### Study area and dataset

**Fig 4:**
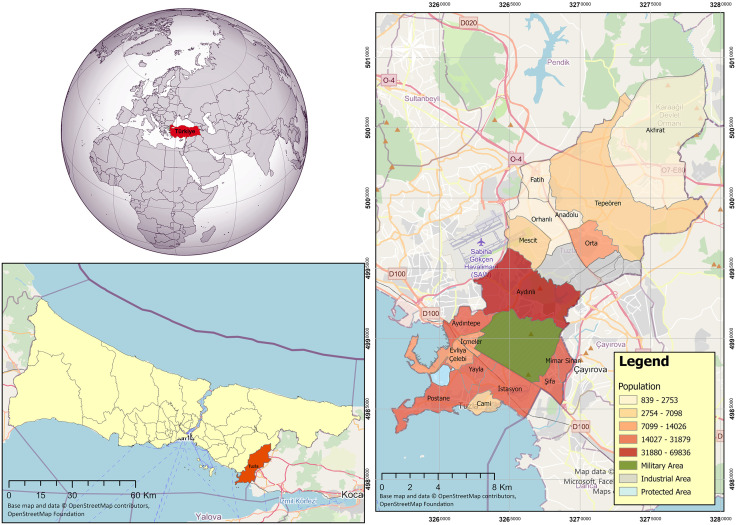
The study area, Tuzla district, İstanbul, Türkiye (Base map and data from OpenStreetMap and OpenStreetMap Foundation).

The study area is a district located in the easternmost part of the Istanbul Metropolitan Area and neighbouring the Sea of Marmara in the South (Fig 4) It has a total of 17 neighbourhoods and its surface area is approximately 138 km^2^ [32]. The district’s climate varies from cold temperatures to warm. The average daily temperature in January is 7.8°C and in July it is 26.3°C [33]. According to the urban-rural neighbourhood classification in the official urban-rural population statistics published by TurkStat, 11 neighbourhoods are in the densely populated areas category, 5 neighbourhoods are in the intermediate-density areas category and 1 neighbourhood is in the thinly populated areas category [34]. The district, which has had an increasing population trend in recent years, has a population of 301.400 people as of 2024 and the majority of the population resides in the south of the district [35]. In addition to urban residential areas, it has different land use classes, such as rural, industrial, protected, and military areas.

**Fig 5:**
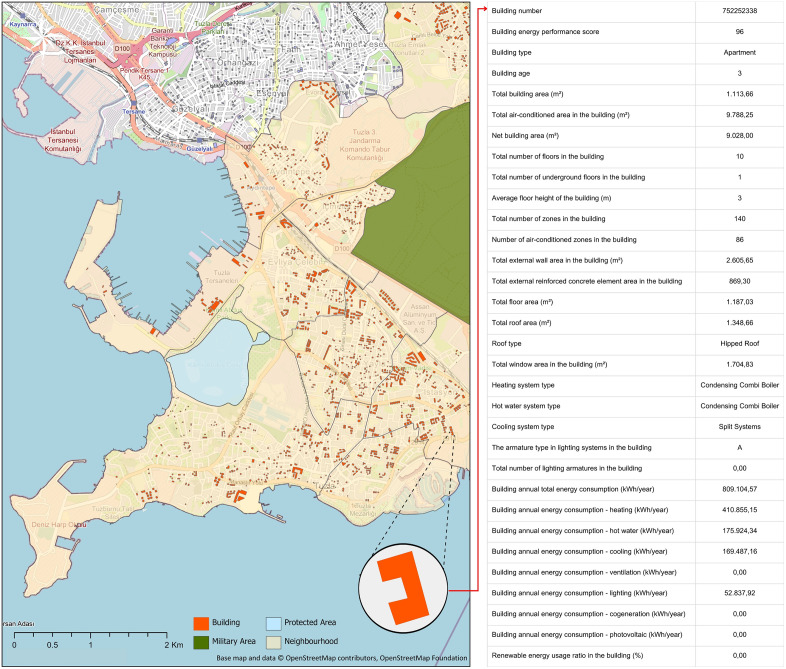
Creating geodatabase for building energy extension of NSDI (Base map and data from OpenStreetMap and OpenStreetMap Foundation).

To develop prediction models of building energy performance scores with ML algorithms, a new GIS-based geodatabase (Fig 5) was created with spatial building data obtained from relevant local governments and building energy data obtained from the energy performance certificates [36].The created geodatabase contains 4637 buildings and 31 variables describing the building energy information detailed in Table 1.

**Table 1:** Building energy dataset features for modelling.

Feature	Data Type	Unit
Building energy performance score	Float	Number
Building type	Categorical	–
Building age	Integer	Number
Total building area	Float	(m²)
Total air-conditioned area in the building	Float	(m²)
Net building area	Float	(m²)
Total number of floors in the building	Integer	Number
Total number of underground floors in the building	Integer	Number
Average floor height of the building	Float	m
Total number of zones in the building	Integer	Number
Number of air-conditioned zones in the building	Integer	Number
Total external wall area in the building	Float	(m²)
Total external reinforced concrete element area in the building	Float	(m²)
Total floor area	Float	(m²)
Total roof area	Float	(m²)
Roof type	Categorical	–
Total window area in the building	Float	(m²)
Heating system type in the building	Categorical	–
Hot water system type in the building	Categorical	–
Cooling system type in the building	Categorical	–
The armature type in lighting systems in the building	Categorical	–
Total number of lighting armatures in the building	Integer	Number
Building annual total energy consumption	Float	kWh/year
Building annual energy consumption – heating	Float	kWh/year
Building annual energy consumption – hot water	Float	kWh/year
Building annual energy consumption – cooling	Float	kWh/year
Building annual energy consumption – ventilation	Float	kWh/year
Building annual energy consumption – lighting	Float	kWh/year
Building annual energy consumption – cogeneration	Float	kWh/year
Building annual energy consumption – photovoltaic	Float	kWh/year
Renewable energy usage ratio in the building	Float	Percentage (%)

### Ensemble learning algorithms

In this study, DT-based ensemble learning algorithms are adapted and the performance of these algorithms in predicting energy performance scores are comparatively evaluated. Ensemble learning is an approach that aims to achieve more accurate and stable predictions by combining multiple models. To overcome the prediction limitations of a single model, predictions from different algorithms or different versions of the same algorithm are combined. Namely, instead of a single prediction model, a series of models are trained, and the results of these models are combined to improve the final prediction performance. Ensemble learning algorithms can generally deal with outliers without greatly affecting overall predictive performance [37,38]. The idea behind an ensemble learning structure could be in two different ways: (a) if the ML algorithm generates multiple learners by randomly creating different subsets from the training dataset and combining the results of these learners, this method is called Bootstrap Aggregating (Bagging). Predictions from different learners are combined and averaged to obtain the final prediction. The most popular bagging-based ensemble learning algorithm is RF; (b) if the ML algorithm tries to continuously improve its prediction performance by repeatedly training multiple learners with the same training dataset, this method is called Boosting [39]. Each new learner focuses on the failed predictions of the previous learner and aims to correct these errors. Thus, each new model compensates for the weaknesses of the previous one, increasing the overall performance. The most popular boosting-based ensemble learning algorithms may be GBM, XGBoost, and LightGBM.

RF is one of the popular ensemble learning algorithms based on decision trees developed by Breiman and widely used for regression and classification models [40]. In the modelling process with RF, a decision forest is created by combining a large number of uncorrelated decision trees created independently of each other. By combining the predictions made by each of the trees in the created decision forest, a more accurate prediction is made [41]. It is also expressed as a combination of Bagging and Random Subspace methods, where fewer variables are randomly selected from all variables in the dataset and the variables that perform the best division at the nodes in the decision trees are selected [42]. In the process of developing decision trees in the decision forest, dataset subsets are created by random sampling from the original training dataset containing N number of samples and M number of variables. 2/3 of the subsets are used to create the decision trees while the remaining 1/3, known as Out-of-Bag (OOB) samples, are utilised to analyse the performance of each tree. In the RF algorithm, since decision trees are trained on bootstrap samples, about 1/3 of the training dataset is left out and not utilised in the creation of that tree. These OOB samples serve as a built-in validation set to obtain an unbiased prediction of the trained model’s error without requiring an external validation dataset. At each node of the decision trees, m variables are randomly selected from the M variables in the training dataset. The determined m value is kept constant during the modelling process [43].

GBM is one of the algorithms with high modelling performance for regression and classification models proposed by Freidman [44]. It could also be considered as a type of AdaBoost algorithm that can be flexibly adapted to regression and classification models. GBM is based on the development of a stronger prediction model by combining weak learners such as decision trees [45]. Unlike the RF algorithm, decision trees are interdependent. In the GBM algorithm, a series of weak learners in the form of a single predictive model are trained. The prediction errors for each model in the series are calculated and the prediction errors of the previous model in the series are optimised. This modelling process is performed iteratively, and the model’s prediction error can improve with each iteration. Prediction errors are determined by differentiable loss functions. GBM uses the gradient descent method to improve predictions by minimizing the loss function. This method, which is frequently used in optimization problems, provides the calculation of the minimum point of the loss function and determines how to improve modelling success by iteratively updating the model parameters with the slope information of the function [46,47].

XGBoost is a special version of the GBM algorithm developed by Chen and Guestrin, optimized to improve speed and prediction performance, scalable and integrable to different platforms [48]. The biggest difference compared to the GBM algorithm is the standardisation of the loss function. While GBM utilises only the first-order derivatives (gradients) of the loss function, XGBoost employs a second-order Taylor expansion, as shown in Formula (1), to optimize the loss function more accurately by incorporating gradients and Hessians. In contrast, when GBM encounters a negative value in the loss function, it stops pruning trees, which may limit further optimisation [49].


$$\begin{array}{c} L^{\left(t\right)}=\;\sum_{i=\text{1}}^{n}\left[l\left(y_{i},{\hat{y}}_{i}^{\left(t-\text{1}\right)}\right)+g_{i}f_{t}\left(x_{i}\right)+\frac{\text{1}}{\text{2}}h_{i}f_{t}^{\text{2}}\left(x_{i}\right)\right]+\;\Omega \left(f_{t}\right) \end{array}$$ (1)

Where $L^{\left(t\right)}$ is the total loss for iteration t, $\left(y_{i},{\hat{y}}_{i}^{\left(t-\text{1}\right)}\right)$ is the loss between the $y_{i}$ (actual value) and the prediction obtained from the previous iteration ${\hat{y}}_{i}^{\left(t-\text{1}\right)}$, $g_{i}$ is the first-order gradient, $h_{i}$ is the second-order derivative (Hessian), $f_{t}\left(x_{i}\right)$ is the prediction for the i−th data point produced by the new decision tree added at iteration t and $\Omega \left(f_{t}\right)$ is the regularisation term that penalizes model complexity to prevent overfitting.

XGBoost splits the tree up to the specified maximum tree depth and performs backward pruning. Also, XGBoost utilises a level-wise tree growth strategy where the algorithm searches for the best tree growth depth [39,50]. On the other hand, the most important factor in the success of XGBoost is its scalability in all scenarios. It can work ten times faster than other ML algorithms. XGBoost, which is effective against overfitting, is based on increasing the efficiency of memory resources and processing time with its parallel and distributed computing characteristics [51].

LightGBM was developed in 2017 as part of Microsoft’s Distributed Machine Learning Toolkit project [52]. It is proposed to optimize the inefficiency of gradient boosting-based models when dealing with high dimensional variables and big datasets. It stands out due to its properties such as parallel computing and low memory consumption and offers high performance and low training time in modelling processes with big datasets [53]. It transforms continuous variables in the dataset into discrete variables to minimize the computational cost [54]. While most ensemble learning algorithms grow decision trees according to a level-wise growth strategy, LightGBM utilises a leaf-wise growth strategy. In this growth strategy, the decision trees within the ensemble grow by focusing on leaf nodes. This strategy neglects the stability of the decision trees and splits leaf nodes at each iteration, increasing the learning rate while focusing on reducing the prediction error. In each split, it chooses the split that minimises the prediction error, which allows the model to learn more effectively [55]. Also, LightGBM uses Gradient-based One Side Sampling (GOSS) and Exclusive Feature Bundling (EFB) approaches to overcome the limits related to the conventional histogram-based method performed in gradient boosting-based algorithms [56,57].

### Model performance metrics

There are various performance metrics to measure the predictive success of classification and regression models developed with ML algorithms. Considering regression models, commonly used performance metrics are Mean Absolute Error (MAE), Mean Absolute Percentage Error (MAPE), Root Mean Squared Error (RMSE) and R-Square (R^2^). While low values of MAE, MAPE and RMSE metrics indicate higher prediction performance, high values indicate lower prediction performance. The R² metric measures how much of the total variance in the dependent variable of the model can be explained by the independent variables in the model and has a value in the range of 0–1. The value of the R² metric being close to 1 indicates that the model explains a large proportion of the variance in the dependent variable and demonstrates high predictive performance. The basic equations for calculating these performance metrics are given below [24,58–61].


$$\begin{array}{c} MAE=\frac{\text{1}}{n}\sum_{i=\text{1}}^{n}\left|y_{i}-\hat{y_{i}}\right| \end{array}$$ (2)

$$\begin{array}{c} MAPE=\frac{\text{1}\text{0}\text{0}}{n}\sum_{i=\text{1}}^{n}\frac{\left|y_{i}-\hat{y_{i}}\right|}{y_{i}} \end{array}$$ (3)

$$\begin{array}{c} RMSE=\sqrt{\frac{\text{1}}{n}\sum_{i=\text{1}}^{n}{\left(y_{i}-\hat{y_{i}}\right)}^{\text{2}}} \end{array}$$ (4)

$$\begin{array}{c} R^{\text{2}}=\text{1}-\frac{\sum_{i=\text{1}}^{n}{\left(y_{i}-\hat{y_{i}}\right)}^{\text{2}}}{\sum_{i=\text{1}}^{n}{\left(y_{i}-\overset{―}{y_{i}}\right)}^{\text{2}}} \end{array}$$ (5)

In the equations, $y_{i}$ denotes the actual values of the dependent variable in the model, $\hat{y_{i}}$ denotes the predicted values of the dependent variable, $\overset{―}{y_{i}}$ denotes the mean of the actual values of the dependent variable, and n denotes the test data size used in the performance measurement, where i=1,2,3,…,n.

## Results

### Developing building energy performance score prediction models

To develop ML-based prediction models for building energy performance scores and to measure their performance, the initial dataset was first split into 70% training (3245 building) and 30% testing (1392 building) via random sampling. As the obtained dataset contained complete records for all variables, no missing data handling was required. As part of the data pre-processing step, however, all the categorical variables listed in Table 1 were converted into numerical values in order to prepare the dataset for modelling. On the other hand, when developing prediction models with RF GBM, LightGBM and XGBoost algorithms, there is a set of hyperparameters for each algorithm. For high-performance prediction models, determining the best values for these hyperparameters is of great importance [25,39,60,62,63]. Therefore, the best hyperparameter values for the final prediction models should be obtained. In this study, the tuned values of the hyperparameters for each ML algorithm were determined with the grid-search approach with 10-fold cross-validation. Hyperparameter tuning operations were performed using the “caret” package of the open source R software [64]. The tuning ranges and tuned values applied for the hyperparameters are summarised in Table 2. Then, final prediction models were developed with tuned hyperparameter values. In the models, the building energy performance score was defined as dependent, and all other features were defined as independent variables. All of the prediction models were developed using relevant packages of R software and training dataset [65–68].

**Table 2:** Hyperparameter tuning ranges and best values determined for parameters.

Model	Parameter	Range	Tuned Value
RF	mtry	5, 10, 15	15
	ntree	50, 100, 150	150
GBM	n.trees	50, 100, 150	150
	interaction.depth	5, 6, 7	7
	shrinkage	0.01, 0.05, 0.1	0.1
	n.minobsinnode	10, 15, 20	10
LightGBM	learning_rate	0.01, 0.05, 0.1	0.1
	num_leaves	10, 20, 30	30
	max_depth	5, 6, 7	7
	min_child_samples	10, 20, 30	30
	subsample	0.7, 0.8, 0.9	0.9
	colsample_bytree	0.7, 0.8, 0.9	0.9
XGBoost	nrounds	50, 100, 150	150
	eta	0.01, 0.05, 0.1	0.1
	max_depth	5, 6, 7	7
	min_child_weight	10, 20, 30	10
	colsample_bytree	0.7, 0.8, 0.9	0.9
	subsample	0.7, 0.8, 0.9	0.9
	gamma	0.01,0.05,0.1	0

### Evaluating model performance

**Fig 6:**
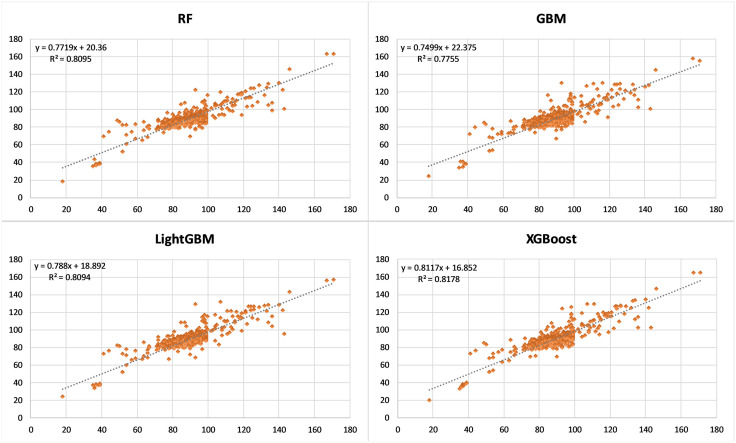
Scatterplots between predicted and actual values for building energy performance score.

The predictive performance of all developed models has been evaluated separately on the test dataset with each of the metrics explained in Table 1. Considering the results, the lowest MAE (2.776) and MAPE (3.264) values were achieved in the RF model. The lowest RMSE (5.153) and the highest R^2^ (0.818) values were achieved in the XGBoost model. Although the results are close to the other models, the lowest modelling performance was observed for the GBM model in terms of all metrics (Table 3). Also, the relationship between the predicted and actual building energy score values in the models was examined with the scatterplots as shown in Fig 6. It was observed that the data distributions were quite similar in all models. The results clearly showed that ML algorithms could be used to predict building energy performance scores.

**Table 3:** Performance of the building energy performance score prediction models.

Model	MAE	MAPE	RMSE	R^2^
RF	2.776	3.264	5.292	0.808
GBM	3.561	4.147	5.731	0.774
LightGBM	2.985	3.491	5.276	0.809
XGBoost	2.886	3.369	5.153	0.818

Subsequently, the statistical significance of the differences in performance among the prediction models was evaluated using the Friedman Test, a non-parametric test commonly used to compare more than two paired groups (models). The test statistic (χ²) for the Friedman Test was calculated as 554.52, with a p-value of < 0.0001. This situation indicates a statistically significant difference in prediction errors among the compared models. Therefore, following the Friedman test, the Nemenyi post-hoc test was conducted to identify which model pairs indicated statistically significant differences. Considering the test results in Table 4, statistically significant differences were observed between all model pairs with p-values less than 0.05. The test results, consistent with the performance metrics, indicate that the RF model indicates a significantly different performance compared to the others, while the GBM model shows a distinct error behaviour relative to the other models. In the comparison between the LightGBM and XGBoost models, the p-value was calculated as 0.8644, indicating that there is no statistically significant difference between the two models. This means that these models indicate similar error distributions.

**Table 4:** The result of Nemenyi post-hoc test for prediction models.

Model Comparison	q value	p-value	Significance
RF – GBM	33.280	<0.0001	***
RF – LightGBM	16.837	<0.0001	***
RF – XGBoost	15.737	<0.0001	***
GBM- LightGBM	16.443	<0.0001	***
GBM – XGBoost	17.543	<0.0001	***
LightGBM – XGBoost	1.100	0.8644	

Signif. codes: *** p < 0.001, ** p < 0.01, * p < 0.05.

The performance of the XGBoost model is partly due to its use of a second-order Taylor expansion of the loss function. Including both first- and second-order gradients enables the model to optimise more accurately during tree construction, resulting in lower prediction errors. XGBoost also supports parallel processing, enabling the simultaneous calculation of tree structures and significantly reducing training time, particularly for large datasets. These characteristics make XGBoost accurate and computationally efficient, which explains why it produces competitive outcomes compared to the other prediction models in this research.

**Fig 7:**
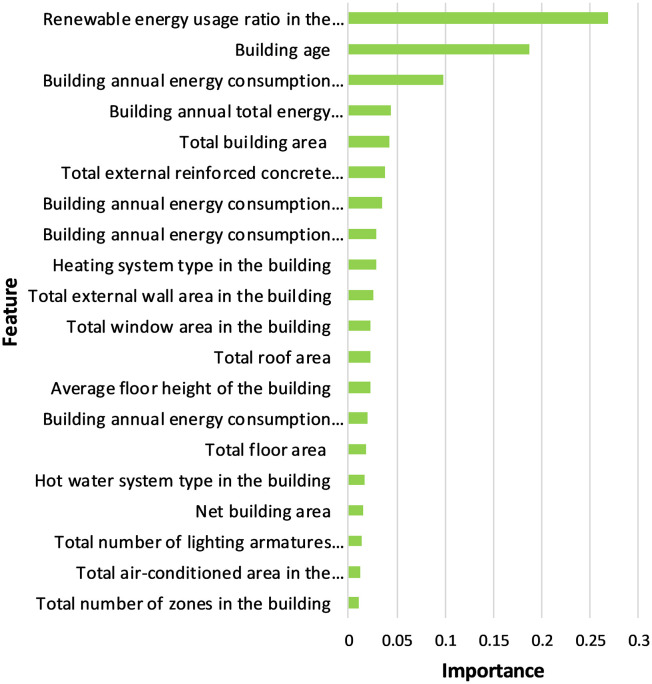
Feature importance of top 20 features in XGBoost model.

On the other hand, feature importance levels were examined for the XGBoost model, which is one of the most successful models. Fig 7 shows the importance levels of the top 20 features that made the greatest contribution to the development of the model. It was observed that features such as renewable energy usage ratio in the building, building age, building annual energy consumption (heating), building annual total energy consumption, total building area, total external reinforced concrete element area, heating system type, building annual energy consumption (lighting), building annual energy consumption (cooling), total external wall area are the foremost features (Fig 7).

## Discussion and conclusion

The buildings are responsible for a significant portion of global energy consumption and GHG emissions. Consequently, enhancing building energy efficiency has become a cornerstone of international climate policy. The Paris Agreement acknowledges energy labelling as a pivotal strategy in achieving climate targets [69]. As outlined in Nationally Determined Contributions (NDCs) defined in the Enhanced Transparency Framework (ETF), countries are obligated to report on their actions and progress towards emission reductions, commencing from 2024 [70]. On the other hand, the 2024 OECD Global Survey on Buildings and Climate reports that over 61% of countries have implemented mandatory energy performance certification systems, with 89% enforcing energy-efficiency codes and 86% offering financial incentives such as subsidies and low-interest loans [71]. However, the majority of countries have not yet developed an information management system to oversee information related to EPB associated with NSDI. Moreover, it appears that EPB has not calculated for the majority of buildings in various countries.

This paper presents a holistic methodology for the ML-based prediction of the EPBs through the use of enhanced NSDI. The novel approach that integrates ensemble ML algorithms with enhanced NSDI is proposed to predict the overall energy performance score of buildings in Türkiye. Following the enhancement of the existing NSDI (i.e., TUCBS) with information from energy performance certificate, specifically the extended energy performance data model, a geodatabase was implemented. The developed geodatabase has been populated with information on 4637 buildings. Subsequently, the database is utilised to develop energy performance prediction models with ensemble ML algorithms, namely RF, GBM, LightGBM and XGBoost in order to estimate the overall performance scores of the buildings lacking energy performance certificates. The findings of the model demonstrated robust predictive accuracy, achieving an R² of 0.818 (XGBoost), with performance metrics of RMSE = 5.153, MAE = 2.886, and MAPE = 3.369, while the lowest MAE = 2.776 and MAPE = 3.264 values were achieved in the RF model. The test results show that, when selecting a model, it is important to consider not only performance metrics, but also statistical comparisons. The test results reveal that LightGBM and XGBoost models have similar error distributions, whereas RF and GBM models perform significantly differently to other models. It can be posited that this approach utilises a spatial and building energy-rich dataset to develop high-accuracy predictive models. The analysis shows that the top five attributes are the amount of renewable energy used by a building, how old the building is, how much energy is used by the building in a year (for heating), how much energy the building uses in total every year, and the total area of the building. It is important to note that two of the top five attributes (i.e., annual energy consumption for heating and annual total energy consumption) are not included in the NSDI of Türkiye (i.e., TUCBS) and they are proposed to be added as an extension in this paper.

There are a number of limitations of this study. Firstly, the proposed machine learning framework has only been tested in one district, which may give rise to concerns about its generalizability. The question of the model’s consistency in other regions or on an international scale remains unresolved, particularly in regions characterised by distinct climatic conditions, architectural standards, or levels of data availability. The model’s applicability should be further tested through evaluation in diverse geographic areas, given the potential significant influence of regional characteristics, including building typologies and regulatory frameworks, on energy performance. Secondly, a proportion of the buildings within the designated study area are not accompanied by energy performance certificates. This deficiency has the potential to impede the accessibility of data and introduce biases into the training set. This issue is mitigated by the utilisation of certified buildings representing a range of energy classes. However, techniques such as synthetic data generation or imputation were not employed. The exploration of these methodologies has the potential to enhance representativeness and mitigate the presence of bias. Additionally, the dataset utilised in this study is predominantly composed of regional data and does not incorporate behavioural variables such as occupant habits, usage patterns, or adaptive comfort strategies, which have the capacity to influence energy consumption. Furthermore, the scope of the algorithmic comparison was limited to tree-based models (RF, GBM, LightGBM, XGBoost), which were selected due to their strong performance in handling tabular data, interpretability, and robustness against overfitting. However, no comparison was conducted with other algorithm families, such as neural networks or support vector regression, which could potentially offer different levels of predictive accuracy. Future research may encompass a broader spectrum of algorithms and additional contextual and behavioural variables. This may enhance the robustness, scalability, and external validity of the proposed framework.

The development of a comprehensive energy model for buildings has facilitated the integration of energy data with numerous datasets within the scope of NSDI in this research. Consequently, while continuing to depend on the fundamental geographic information infrastructure, considerably more comprehensive and functional data on energy performance at the building scale has been include in NSDI. The extended data model has the potential to enhance the efficacy of building energy management and facilitate the acquisition of substantial and informative data for spatial analyses, decision support systems and city information systems. This, in turn, will underpin the development of applications and policies regarding building energy efficiency in smart cities by means of evidence-based decision-making.

The predictive modelling of energy performance score offers a wide array of advantages that extend across multiple domains of policy, urban planning, and sustainability. For instance, the predicted energy performance based on SDI integration has the potential to inform the retrofitting priorities of authorities, enabling them to target incentives or supports towards the least efficient buildings. This is is consistent with Türkiye’s “Energy Efficiency 2030 Strategy and II. National Energy Efficiency Action Plan (2024-2030)”. Furthermore, it has the capacity to facilitate the conceptualisation of carbon penalty schemes or performance-based taxation systems, particularly in scenarios where energy performance certificate coverage is limited and compliance monitoring is challenging. The model outputs can also be integrated into urban planning dashboards or GIS-based decision-support tools, allowing stakeholders to visualise spatial patterns of energy performance, simulate the impact of various policy interventions, and optimize investment strategies at the district or city scale. Furthermore, the proposed approach can function as a guideline for the selection of priority zones for transformation projects (e.g., urban transformation), thereby facilitating a more efficient allocation of resources, time, cost and workload. Finally, the approach can contribute towards the realisation of sustainability and climate objectives by facilitating GHG reductions, thereby reinforcing Türkiye’s commitment to international goals such as the Paris Agreement, Sustainable Development Goals and INSPIRE Directive, and by enhancing the nation’s integration with European and global climate governance systems.
